# Baseline Characteristics of Participants in the Alberta Cancer Exercise Hybrid Effectiveness–Implementation Study: A Wake-Up Call for Action

**DOI:** 10.3390/cancers17050772

**Published:** 2025-02-24

**Authors:** Margaret L. McNeely, Shirin M. Shallwani, Tanya Williamson, Christopher Sellar, Elaine Gobeil, Anil Abraham Joy, Harold Lau, Jacob Easaw, John Sexsmith, Kerry S. Courneya, S. Nicole Culos-Reed

**Affiliations:** 1 Department of Physical Therapy, University of Alberta, 2-50 Corbett Hall, Edmonton, AB T6G 2G4, Canada; sshallwa@ualberta.ca (S.M.S.); csellar@ualberta.ca (C.S.); egobeil@ualberta.ca (E.G.);; 2 Department of Oncology, University of Alberta, 11560 University Ave., Edmonton, AB T6G 1Z2, Canada; 3 Faculty of Kinesiology, University of Calgary, 2500 University Dr. NW, Calgary, AB T2N 1N4, Canada; willt@ucalgary.ca (T.W.); nculosre@ucalgary.ca (S.N.C.-R.); 4 Department of Medical Oncology, Cross Cancer Institute, 11560 University Ave., Edmonton, AB T6G 1Z2, Canada; jay.easaw@ahs.ca; 5 Arthur J. E. Child Comprehensive Cancer Centre, 3395 Hospital Drive NW, Calgary, AB T2N 5G2, Canada; hlau@ucalgary.ca; 6 Department of Oncology, University of Calgary, 2500 University Dr. NW, Calgary, AB T2N 1N4, Canada; 7 Faculty of Kinesiology, Sport, and Recreation, University of Alberta, 1-113 University Hall, Edmonton, AB T6G 2H9, Canada; kerry.courneya@ualberta.ca

**Keywords:** cancer survivorship, exercise, physical activity, quality of life, supportive care, implementation, knowledge translation

## Abstract

**Background:** Alberta Cancer Exercise (ACE) is a hybrid effectiveness–implementation study evaluating a cancer-specific community-based exercise program across urban sites in Alberta, Canada. The purpose of this paper is to describe the baseline characteristics of participants. **Methods:** Adults with any type and stage of cancer, who were undergoing cancer treatment or up to three years post treatment completion, were eligible. ACE was delivered in person at 18 sites across 7 cities in Alberta, with video conferencing introduced during the COVID-19 pandemic. Participants took part in 60 min of mild-to-moderate intensity exercise twice weekly for a 12-week period and were encouraged to increase overall physical activity. **Results:** From January 2017 to February 2023, 2570 individuals enrolled. Participants were a mean age of 57.8 years, 71.3% were female, 45.4% had breast cancer, and 49.4% were undergoing cancer treatment. At baseline, only 22.4% of participants self-reported meeting recommended physical activity levels, 66.0% were overweight/obese, and 71.4% reported one or more comorbidities. Most participants were below normative levels for the six-minute walk and 30 s sit-to-stand tests, and 75.9% reported fatigue. **Conclusion:** Participants were largely inactive, unfit, and symptomatic. ACE attracted more females and individuals with breast cancer but was otherwise representative of the Alberta cancer population.

## Simple Summary

Exercise is an effective intervention that can optimize the health and wellbeing of individuals with cancer and possibly reduce rates of cancer recurrence and secondary cancers. This paper reports the baseline characteristics of participants taking part in a cancer-specific community-based exercise effectiveness–implementation study called Alberta Cancer Exercise (ACE). The exercise program was offered twice weekly for a 12-week period, and was tailored to the individual’s fitness level, and personal fitness or lifestyle goals. A total of 2570 individuals were screened and had measurements taken at the start of the study to evaluate exercise safety, physical activity and fitness levels, and cancer-related symptoms. Participants were then referred to a suitable community exercise program. Due to its large sample size, ACE is a resource that will help to inform the implementation of exercise programs in real-world settings for diverse groups of individuals with cancer.

## 1. Introduction

There is a large and increasing evidence base supporting the benefits of exercise for people living with and beyond cancer [1]. Exercise is a relatively low-cost and safe intervention that can reduce treatment-related side effects and benefit physical functioning, health-related fitness, and psychosocial wellbeing [1,2]. The evidence on the benefits of exercise is particularly strong for symptoms of fatigue, anxiety, and depression [2]. Moreover, exercise is associated with a 40% to 50% reduced disease-specific and overall mortality among individuals diagnosed with breast, colorectal, or prostate cancer [2]. While individuals with cancer report a high interest in exercise, a minority self-report meeting recommended levels of physical activity [3,4]. Barriers to exercise include cancer-related symptoms, time, and lack of motivation [5].

Current guidelines recommend the integration of exercise into cancer care [6,7] as a means to support exercise adoption [1]. Research studies examining implementation of community-based exercise programs demonstrate short-term effectiveness yet lack data on long-term effectiveness [8,9]. Commonly reported issues include low attendance at exercise sessions (e.g., <70%) and less than optimal completion rates (40% to 68%) across sites for physical fitness and patient-reported outcomes [9,10,11]. In Canada, a national cross-sectional study was conducted including 179 facilities providing cancer medical services. Twenty sites reported having a formal oncology rehabilitation program [12]; however, only eight offered some exercise-related programming. To date, no large-scale publicly funded programs exist in Canada, nor strategies to bridge individuals with cancer to resources and programs that are in place [13]. Reported barriers to formal oncology rehabilitation programs include a lack of funding and access to space and equipment [12].

To address these challenges, we conducted a large-scale hybrid effectiveness implementation study across urban sites in Alberta. The Alberta Cancer Exercise (ACE) program aims to deliver high quality cancer-specific community-based exercise programming for individuals living with and beyond cancer. The primary aim of this paper is to present the baseline medical, demographic, lifestyle, symptom, and fitness characteristics of the participants taking part in the study. A secondary aim is to compare ACE participants to the Alberta population and their baseline results to standardized fitness norms.

## 2. Methods

### 2.1. Study Design and Participant Recruitment

The study design and methods have been previously described [14]. Briefly, the ACE study opened in January 2017 and completed recruitment in February 2023. Our design included an integrated knowledge translation strategy involving individuals with lived experience of cancer in the design and ongoing delivery of ACE. Co-design features proposed by patient partners and approved by our medical advisors included (1) eligibility of adults with all types and stages of cancer and (2) the ability of individuals with cancer to self-refer to the program [15]. Moreover, our patient partners expressed a preference for programming that started with low intensity exercise and accommodated varying levels of ability.

Oversight and coordination for ACE was conducted through two hub sites in the province of Alberta: one in the north at the University of Alberta (Edmonton) and one in the south at the University of Calgary (Calgary). For this large-scale hybrid effectiveness–implementation study, the hub clinical exercise physiologists (CEPs) with expertise in cancer led the recruitment, referral, and the fitness testing at the community sites. The Health Research Ethics Board of Alberta: Cancer Committee approved the study, and all participants provided written informed consent.

### 2.2. Settings

**Figure 1:**
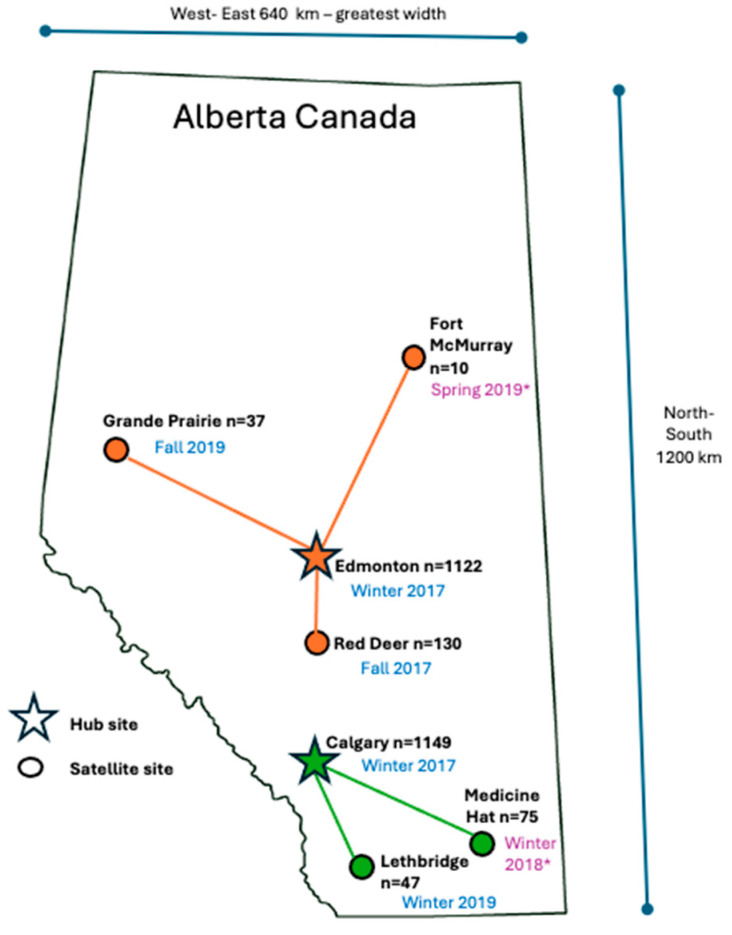
Map of Alberta: ACE roll-out timeframes and numbers for hub and satellite sites. * Sites discontinued after onset of COVID-19 pandemic in March 2020.

ACE 12-week sessions were offered three times a year, with winter, spring, and fall start dates. Participants referred after the entry deadline for a given session were placed on the waitlist for the next session. Prior to the coronavirus disease (COVID-19) pandemic, the exercise programming was offered in seven Alberta cities (Calgary, Edmonton, Fort McMurray, Grande Prairie, Lethbridge, Medicine Hat and Red Deer). Programming was rolled out from January 2017 to September 2019 to include a final total of 18 sites: 6 YMCAs, 6 municipal fitness centers, 3 Wellspring Alberta locations (a non-profit cancer support organization), and 3 academic fitness facilities (University of Calgary, University of Alberta and Lethbridge College) (Figure 1). With the onset of COVID-19 in March 2020, exercise testing and programming shifted to video conferencing for the spring 2020 session. Official in-person classes resumed in Edmonton, Calgary, and Red Deer in September 2021, and in Lethbridge in Winter 2022. Virtual group exercise class options continued to be offered through Calgary, Edmonton, Grande Prairie, and Red Deer sites.

### 2.3. Eligibility Criteria

Eligebility criteria included the following:

(1) A diagnosis of cancer of any type and stage;
(2) Eighteen years of age or older (individuals who were 17 years of age were considered if approved by their oncologist);
(3) Able to participate in mild to moderate levels of exercise as determined by the CEP and/or healthcare professional;
(4) The time period relative to diagnosis includes prior to treatment, or receiving active cancer treatment (including treatment for palliative intent), or have received cancer treatment within the past three years, or have existing long-term or late presenting effects of their cancer treatment (exceeding three years) if approved by their oncologist (i.e., lymphedema, radiation fibrosis syndrome, mobility deficits);
(5) Provide informed written consent in the English language.

### 2.4. Screening for Safety

Participants provided initial consent for the collection of self-report data as the first stage of screening. The CEP at each of the hub sites performed the initial screening for exercise safety and determined the participant’s appropriateness for community-based exercise (e.g., no contraindications to exercise testing and training; independent in ambulation and transfers) [16,17]. Study consent was collected at the baseline assessment. The CEP was responsible for overseeing baseline objective tests, interpreting results, and triaging the participant to appropriate programming. The CEP consulted with the participant’s oncologist or family physician, as needed, on the need for further evaluation and/or referral to oncology rehabilitation services (e.g., presence of bone metastases, high resting blood pressure, abnormal heart rhythm, cognitive issues). In 2018, to accommodate participants with complex presentations due to active cancer, metastatic disease, or high symptom-burden, programming to support closer monitoring and supervision by a CEP/physical therapist was instituted.

### 2.5. Exercise Intervention

Using a pragmatic approach, the instructor at the respective community site selected the most appropriate mode of exercise delivery based on their preference, and available space and resources. Options included a group circuit-type class in a studio/virtual setting or group personal training sessions in the site’s fitness center. Class sizes ranged from 8–15 participants. The program included options for low-to-moderate intensity exercise, with the overall exercise volume and intensity slowly progressing over the 12-week program duration. An ACE-trained exercise specialist led both the in-person and virtual classes. For virtual sessions, all participants had cameras on and were ‘in view of the instructor’ for safety during the class. A second ACE-trained exercise specialist was present at virtual ACE sessions for safety and participant assistance. Participants were provided with exercise behavior change education that included goal-setting and activity monitoring to support efforts to increase overall physical activity towards guideline levels [18]. Further details on the ACE intervention are provided in a Template for Intervention Description and Replication (TiDIER) checklist (Supplementary Table S1).

### 2.6. Outcomes to Assess Effectiveness of Programming

As previously described [14], the ACE CEPs conducted the baseline fitness assessments at the university sites or at the respective fitness facilities offering the programming. Prior to COVID-19, the ACE hub staff traveled to the respective smaller cities in the north (Red Deer, Fort McMurray, Grande Prairie) and south (Lethbridge, Medicine Hat) of Alberta to conduct the baseline and 12-week assessments. During the COVID-19 pandemic, fitness testing was conducted virtually when in-person testing was not possible, and virtual testing continued after the pandemic for individuals residing in the smaller cities and was offered as an option for those choosing to participate in virtual exercise classes. Reflective of our implementation focus, adaptations were made to the selected patient-reported and fitness testing outcomes over time.

### 2.7. Self-Reported Questionnaires Collected Online at Baseline for All Participants

Demographic and medical data were collected through self-report, the Alberta Cancer Registry, and abstracted from the electronic medical chart. A series of questionnaires were completed online for both in person and virtual programming including the revised Edmonton Symptom Assessment Scale (ESAS-r) [19], the Functional Assessment of Cancer Therapy-General (FACT-G) [20] and Fatigue (FACIT-F) scales [21], the modified-Godin Leisure Time Physical Activity Questionnaire [22,23], and the EuroQol EQ5D-5L [24], with a questionnaire on treatment-related impairments added in fall 2019. During the COVID-19 pandemic, both the Lower Extremity Functional Scale [25] and Upper Extremity Functional Scale [26] were added to capture participant physical functioning given the limited number of tests that could be performed virtually. To reduce participant-reported questionnaire burden, the RAND 36-Item Health Survey [27] was discontinued after winter 2020.

### 2.8. In-Person Objective Outcome Measures at Baseline Included

In-person fitness assessments included resting blood pressure, heart rate and oxygen saturation, height and weight (calculation of body mass index), hip and waist circumference measurements [28], 30 s timed sit-to-stand [29], active shoulder flexion range of motion [30] (flexibility), single-foot balance [31], handgrip strength [32,33,34], and the six-minute walk test (6 MWT) [35]. Based on feedback from ACE exercise specialists and CEPs, the single foot balance with eyes closed and measurements of hip and waist circumference were removed from the fitness testing battery after spring 2018 and winter 2020, respectively.

### 2.9. Virtual Objective Outcome Measures Included

Virtual fitness assessments included heart rate (self-measured), two-minute step test (2 min step) [36], 30 s sit-to-stand [29], shoulder flexion range of motion [30] (flexibility), and single-leg balance [31]. Where possible, data were collected on blood pressure and body weight (if home devices were available).

Additional tests were performed where equipment, time, and resources allowed including: (i) one-repetition maximum bench press (in-person) for upper body muscular strength; (ii) one-repetition maximum leg press (in-person) for lower body muscular strength; (iii) sit-and-reach test (in-person) or single-leg sit-and-reach test (virtual) for flexibility; and (iv) plank test for core muscular endurance (in-person and virtual).

### 2.10. Sample Size and Analyses

The original sample size goal for the ACE five-year study was 1000 participants. As the target of 1000 participants was reached at 25 months, in advance of the full program roll-out, the sample size was increased to 2500 [14]. A sample size of 305 participants was calculated, based on data from our prior feasibility RCT, as well as a two-sided significance level of 1% and a power of 90%, assuming a minimally important difference of 10% in the proportion of participants meeting guideline levels for physical activity at one-year follow-up [9].

Study data were collected and managed using REDCap electronic data capture tools hosted and supported by the Women and Children’s Health Research Institute at the University of Alberta [37]. Descriptive analyses (means, standard deviation, frequencies and percentages) were performed to evaluate participant demographic, medical, lifestyle, symptom, and fitness variables by hub site. To inform clinical interpretation, we further classified the participants’ physical activity and body composition data and categorized fitness testing data relative to available normative values for age and biological sex [38,39,40,41,42,43,44]. Fitness testing findings for the 6 MWT (hallway test only), 2 min step, 30 s sit-to-stand, single-leg balance, and shoulder range of motion were dichotomized into one of two categories: (1) below normal levels for age and biological sex or (2) meeting/exceeding normal levels for age and biological sex.

## 3. Results

**Figure 2:**
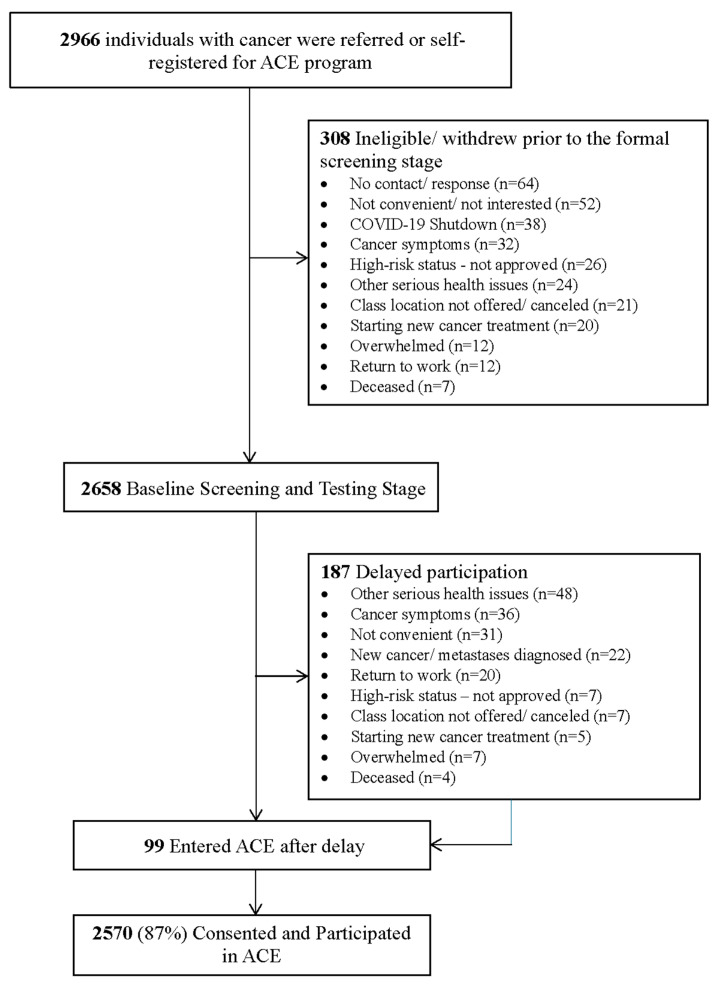
Flow of the participants through baseline assessment in the Alberta Cancer Exercise, Alberta, 2017–2023.

A total of 2966 individuals with cancer were referred or self-referred to the ACE program (Figure 2). Of these, 308 individuals opted not to proceed after the initial registration phase. We conducted baseline screening on 2658 individuals (89.6%) and 2570 (86.6%) participated in the ACE program. Of participants who enrolled, 40% had received a recommendation to ACE by a member of the oncology team (i.e., oncologist, nurse, or cancer rehabilitation professional).

**Figure 3:**
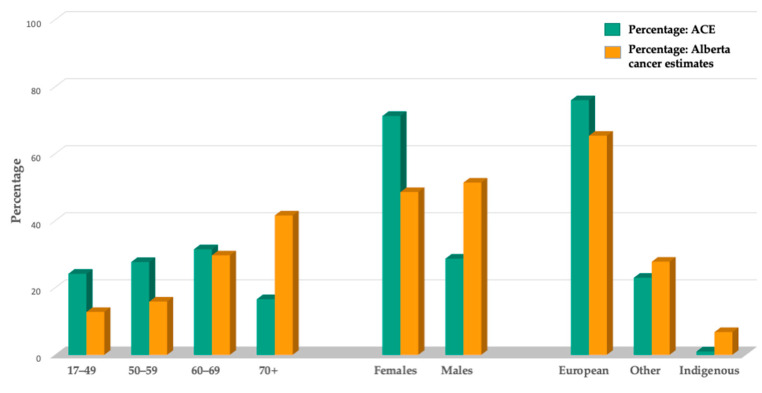
Comparison of demographics relative to Alberta Cancer Exercise and population estimates.

**Figure 4:**
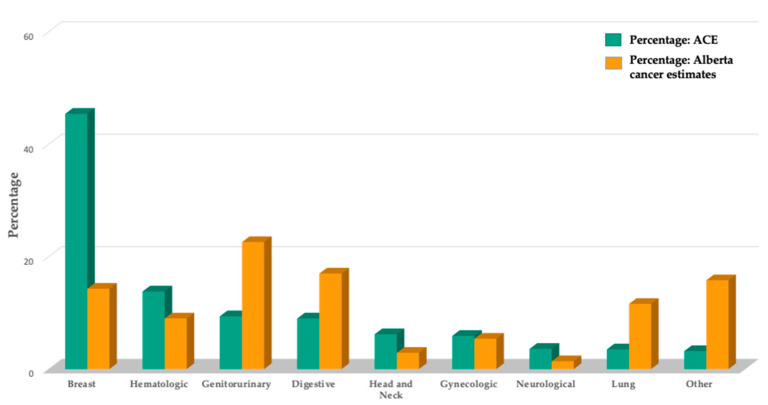
Comparison of cancer tumor types relative to Alberta cancer estimates [46].

On average, ACE participants were 57.8 years of age (SD = 12.0) (Table 1). Relative to the Alberta cancer population, ACE attracted more females, individuals who were younger, and those of European descent (Figure 3) [45,46]. Fewer participants were in the highest income bracket compared to Alberta population estimates. Breast cancer was the most common cancer type (45.4%), followed by hematologic cancers (13.8%) (Table 2). Compared to estimates for Alberta, ACE had a lower proportion of participants with common cancer types such as digestive, genitourinary, and lung cancers (Figure 4) [46]. From an exercise testing and training risk perspective, 28% of participants had high-risk cancers and/or had confirmed metastatic disease. Treatment status was distributed almost evenly between participants on cancer treatment (49.4%) and off treatment (50.6%).

Aligning with population estimates, ACE participants were mainly never smokers (59.9%) and social drinkers (73.2%) (Table 3) [45]. Most participants reported one or more comorbid conditions (71.4%), with arthritis being the most common (45.5%) followed by heart disease (25.7%). On average, participants reported completing 85.6 min (SD 136.6) of physical activity per week and were generally insufficiently active (31%) or sedentary (46.6%).

Supplementary Table S2 provides details on the baseline questionnaire and fitness testing completion rates overall and by north and south hub sites. We had high baseline completion rates across required outcome measures at both hub sites. The baseline completion rate for questionnaires was 99.8% for individuals participating in person and 100% for those taking part in virtual programming. Fitness testing completion rates ranged from a low of 93.3% for the single-foot balance test to a high of 99.6% for the 6 MWT. Primary reasons for non-completion included drop-out prior to baseline fitness testing and pain and safety reasons. Optional tests were completed by approximately one-third of participants, with the plank endurance test being the most completed test (31.7%).

The most common reported treatment-related impairment was cancer-related fatigue (75.9%), followed by arthralgias and myalgias (joint and muscle pain and weakness; 51.9%), and chemotherapy-induced peripheral neuropathy (43.3%). One or more cancer-related symptoms were reported on the ESAS-r questionnaire by 96.9% of participants, with the highest frequency seen for tiredness (89.6%), pain (70.2%), and drowsiness (69.4%) (Table 4). Supplementary Table S3 provides baseline values for self-reported health dimensions (EQ5D-5L), quality of life (FACT-G, FACIT-Fatigue and Rand 36-Item Health Survey), and functional outcomes (Upper Extremity and Lower Extremity Functional Index scores).

**Figure 5:**
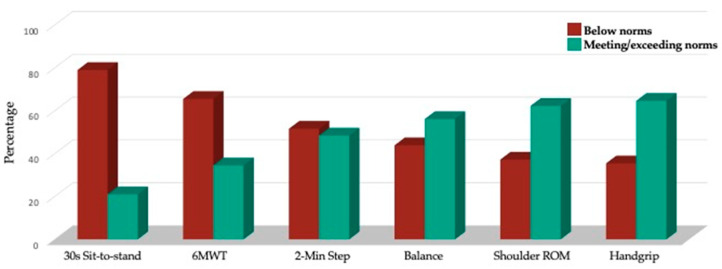
Percentage of participants below versus meeting/exceeding standardized norms for measures of physical fitness.

The ACE participants’ average body mass index was 28.0 (SD = 6.0), with 65.8% falling in the overweight or obese categories (Table 5), rates closely aligning with Alberta population estimates [48]. Most ACE participants were categorized as below standardized normative levels for the 30 s sit-to-stand (*n* = 2551; 79.0% below), 6 MWT (*n* = 780 participants completing the hallway test; 65.5% below norms), and 2 min step (*n* = 455 tested virtually; 51.6% below norms). Fewer participants fell below standardized normative levels for the single-foot balance (*n* = 2560; 43.9%), shoulder flexion range of motion (*n* = 2560; 38.5%), and combined grip strength (*n* = 1746; 33.2%) (Figure 5).

## 4. Discussion

This article describes the baseline characteristics of participants taking part in ACE across urban sites in Alberta, Canada. Distinct from controlled trials of exercise interventions, the data reported here are based on the real-world implementation of a cancer-specific exercise program in community settings. Initial recruitment into the study exceeded expectations, with 1753 participants taking part from January 2017 until March 2020. However, enrollment dropped significantly with the COVID-19 pandemic in 2020, and consequently it took an additional year (total of six years) to reach our sample size of 2500. In line with other implementation studies, ACE attracted a slightly younger sample of individuals with cancer, with a high proportion of females diagnosed with breast cancer [49]. Although breast cancer was highly represented in ACE (45.4%) compared to the Alberta cancer rate (14.2%) [46], this is not surprising given both the evidence supporting exercise in this population [7] and the extensive exercise oncology research involving breast cancer conducted in the province, thus facilitating both self-referral and referral from the oncology teams [50,51,52,53].

A primary finding was that ACE participants were largely inactive, unfit, and symptomatic. Only 22.4% of participants were meeting recommended levels for physical activity, a level substantially lower than the 2020 Canadian population rate of 49.2% [47]. While reported variations exist in the literature on physical activity levels of individuals with cancer [54], our rate is lower than that of a recent Canadian study (28%) [55] and rates reported among survivors in the United States (33%) [56]. Although exercise is known to be effective in addressing physical and psychosocial outcomes both during and following cancer treatment, these low rates of physical activity are not surprising [2]. Our baseline findings provide evidence supporting the high prevalence of impairments and symptoms, barriers known to affect the individual’s motivation to exercise [4,57]. For this reason, we did not exclude participants who self-reported meeting physical activity guideline levels, recognizing that these levels represent minimum standards, and that participants may require exercise support to maintain levels during treatment and/or to ensure successful return to higher levels of usual activities (e.g., sport, work).

Patient research partners have been actively engaged in ACE from inception, as such, we were able to ensure that ACE aligned with the needs and preferences of patients [58]. This included implementing the self-referral option, having cancer-trained community exercise specialists leading classes, and hub CEPs facilitating site referral and registration—aspects identified as critical for program access and adoption [15]. Although successful in enrolling large numbers of participants, we experienced a recruitment challenge relating to underserved populations such as visible minorities, common cancers besides breast cancer, male participants, and older adults. The percentage of participants from visible minorities (22.5%) was below Alberta population estimates reported for all ages (27.8%) [59,60], and those identifying as Indigenous (1.0%) were also underrepresented (6.8%) [59]. A lower rate of males and older adult participants are also commonly reported across community-based exercise programs [49,61], suggesting the need for further work to inform the design and features of exercise programming to be acceptable to these and other underserved populations [7].

The onset of COVID-19 resulted in declines in physical activity levels among individuals with cancer, along with disruptions in cancer rehabilitation and exercise services [62,63]. To mitigate these challenges, the ACE study was adapted through (1) the integration of virtual screening, assessment, and exercise programming; and (2) enhanced infection prevention and control measures for in-person activities. Virtual exercise programming allowed for enhanced flexibility, with online exercise classes offering a broader reach for individuals experiencing cancer-related effects (e.g., neutropenia)—a barrier to community exercise participation reported in our prior feasibility trial [9,64]. Conducting study assessments virtually helped to reduce researcher travel time and costs to the smaller urban sites. However, challenges with virtual programming included fewer options for individuals who required more closely supervised exercise, limited objective data to inform tailoring of programming, lack of access to specialized exercise equipment, and fewer opportunities for socialization [65]. Virtual exercise programming also required more extensive screening of participants for exercise safety, additional personnel for exercise class monitoring, and the need for participant technology support. With the resumption of in-person programming, measures were taken to mitigate the increased risk of infections, including smaller class sizes, masking, social distancing (each participant had a marked exercise space), and enhanced cleaning protocols for space and equipment.

At the time of enrolling in ACE, 27.8% of participants reported metastatic disease and 49.4% were undergoing cancer treatment. As individuals with cancer could self-refer to ACE, our screening process started by first identifying individuals at *high risk* from an exercise testing and training perspective [16]. To guide referral to the appropriate community site, we considered the need for supervision and monitoring given the likelihood of fluctuating and accumulating side effects that could compromise exercise participation and safety [66]. Several frameworks now exist to enable clinical decision-making for the screening, triage, and referral of individuals with cancer to appropriate exercise resources and services [13,66,67]. Screening approaches, while differing across frameworks, are consistent in their emphasis on the individual’s risk, cancer profile, physical or functional status, motivation, and physical activity levels. The complexity of participants enrolling in ACE, along with the low levels of physical activity and fitness seen in this study, support the need for further development and testing to determine their value and the extent that these frameworks can guide clinical practice. Moreover, there is a clear need for investment in exercise programming to improve the overall health of individuals undergoing and recovering from cancer treatment [68].

## 5. Limitations

A limitation of the ACE hybrid effectiveness–implementation study is the lack of a control group to allow for the future investigation of findings relative to usual care. Generalizability of the program, however, is supported by the broad inclusion, clinically relevant outcomes, and community-based implementation approach. Further limitations included the higher education level of participants and the exclusion of individuals who were unable to provide consent in English. While the English language requirement was in place to ensure safety given the delivery in a community-based setting, advances in technology may facilitate future programming in other languages (e.g., virtual classes offered in alternate languages). Our findings are further limited by the high percentage of female participants and those with breast cancer; however, our large sample size will allow us to report data separately for males and for many understudied cancer types.

## 6. Conclusions

Our ACE patient research partners noted the low levels of physical activity and fitness among participants and the high numbers reporting other comorbid diseases—indicators they felt should serve as a “wake-up call” to individuals with cancer. In line with calls by researchers in the exercise oncology field, they recommended “action” on the part of healthcare professionals to support the implementation of exercise programming, as well as counseling and referral practices, into standard cancer care [68,69,70]. Moreover, they suggested the exploration of strategies to support recruitment of representative samples, especially those with prostate and colorectal cancers who may realize benefit from exercise for both supportive care and cancer outcomes.

Notwithstanding these limitations, ACE will be a valuable future resource to inform both the short- and long-term effectiveness of exercise and address key knowledge gaps in the implementation of cancer-specific exercise programs in real-world settings [49,71].

## Figures and Tables

**Table 1:** Demographic characteristics at baseline (*n* = 2570) and Alberta population estimates.

Demographic Characteristic	Total Cohort (*n* = 2570)		Alberta Estimates [46,47]
	Mean/*n*	SD/%	Mean/%
Age years, mean, SD	57.8	12.0	66.9
17 to 39 years	190	7.4%	5.0%
40 to 49 years	433	16.8%	7.8%
50 to 59 years	712	27.7%	15.9%
60 to 69 years	809	31.5%	29.7%
70+ years	426	16.6%	41.6%
Biological sex			
Female	1832	71.3%	48.6%
Male	738	28.7%	51.4%
Population group: ethnic background			
European (White)	1859	72.3%	65.4%
North American—Other	56	2.2%	-
North American—Indigenous	25	1.0%	6.8%
Asian	261	10.2%	27.8%
Multiple ethnicities	158	6.1%	
Latin, Central, South America	34	1.3%	
African	26	1.0%	
Caribbean	13	0.5%	
Oceanian	7	0.3%	
Other	11	0.4%	
Unknown/not reported	120	4.1%	-
Marital status			
Never married	261	10.2%	28.1%
Married or common-law	1858	72.3%	58.9%
Divorced or separated	339	13.2%	8.6%
Widowed	112	4.4%	4.4%
Highest level of education			
No certificate	99	3.8%	9.9%
High school certificate+	724	28.3%	25.3%
Completed university/college	1334	51.9%	55.4%
Completed graduate school	412	16.0%	9.4%
Missing/not reported	1	0%	-
Household Income			
≤ CAD 39,999	398	15.5%	15.1%
CAD 40,000 to CAD 59,999	369	14.4%	12.7%
CAD 60,000 to CAD 99,999	716	27.9%	24.7%
≥CAD 100,000	810	31.5%	47.5%
Missing/not reported	277	10.8%	-

“-“ no data available.

**Table 2:** Cancer and treatment characteristics at baseline (*n* = 2570).

Cancer and Treatment Characteristics	Total Cohort (*n* = 2570)	
	Mean/*n*	SD/%
Number of cancers diagnosed		
Single primary cancer	2392	93.1%
Two primary cancers	161	6.3%
Three+ primary cancers	17	0.7%
Primary Cancer Type *		
Breast	1167	45.4%
Hematologic	355	13.8%
Genitourinary	241	9.4%
Digestive	231	9.0%
Head and Neck	159	6.2%
Gynecologic	152	5.9%
Neurological	93	3.6%
Lung	90	3.5%
Other	82	3.2%
High risk status/confirmed metastatic disease **	714	27.8%
Cancer Treatment Status		
On treatment	1269	49.4%
Off treatment	1301	50.6%
Current cancer treatment—type		
Chemotherapy	477	18.6%
Radiation Therapy	134	5.2%
Hormonal Therapy	570	22.2%
Targeted/Biological	229	8.9%
Other treatment	42	1.6%
Cancer treatments completed—type		
Surgery	1802	70.1%
Chemotherapy	1475	57.4%
Radiation Therapy (RT)	1327	51.6%
Hormonal Therapy	285	11.1%
Targeted/Biological	102	4.0%
Stem Cell Transplant	93	3.6%
Other treatment **	28	1.1%

* When one or more cancers were reported, the primary cancer was based on study eligibility (on treatment or within 3 years of treatment completion). ** High risk: multiple myeloma, head and neck, lung, pancreatic, and primary brain tumors or confirmed metastatic disease to a distant site or organ.

**Table 3:** Lifestyle characteristics at baseline (*n* = 2570) and Alberta population estimates.

Lifestyle Characteristics			Total Cohort (*n* = 2570)		Alberta Estimates [47]
Smoking status	Mean/*n*	SD/%	%		
Never smoker			1540	59.9%	60.0%
Ex-smoker			908	35.3%	24.2%
Current smoker			121	4.7%	15.8%
Not reported	1	0.0%	-		
Drinking status					
Never drinker			337	13.1%	24%
Ex-drinker	271	10.5%			
Social drinker			1880	73.2%	76%
Daily drinker	81	3.2%			
Not reported	1	0.0%	-		
Comorbid Disease—mean number of conditions			1.27	1.11	NR
Number of Comorbid Conditions					
No comorbid conditions			735	28.6%	54.9%
One comorbid condition			886	34.5%	45.1%
Two comorbid conditions	584	22.7%			
Three or more conditions	365	16.1%			
Type of comorbid disease					
Arthritis			1168	45.5%	21.2% *
Heart Disease/HT			659	25.7%	19.9%
Mental Health Issues			388	15.1%	15.9% *
Respiratory Disease			304	11.8%	20.0% *
Metabolic Disease			250	9.7%	7.4% *
Prior Stroke			65	2.5%	2.9% *
Other health condition			426	16.6%	NR
Physical Activity mins/week			85.6	136.6	NR
Physical Activity category					
Active			576	22.4%	49.2 *
Insufficiently Active			796	31.0%	NR
Sedentary	1195	46.6%			

* Canadian population estimates: HT—hypertension “-“ no data available; NR: not reported.

**Table 4:** Self-reported symptoms and impairments at baseline (*n* = 2570).

Cancer Impairments and Symptom Profile		Total Cohort	
		No.	%
Cancer treatment-related impairments	(*n* = 1216) *		
Cancer-related fatigue		923	75.9%
Arthralgias and myalgias		631	51.9%
Chemotherapy-induced peripheral neuropathy		527	43.3%
Body weight management concerns		408	33.6%
Cognitive challenges		409	33.6%
Lymphedema		225	18.5%
Incontinence		206	16.9%
Communication issues		68	5.6%
Cardiac issues		50	4.1%
Symptoms: ESAS Score **	(*n* = 2563)		
No reported symptoms		79	3.1%
Tiredness (lack of energy)		2297	89.6%
Pain		1798	70.2%
Drowsiness		1778	69.4%
Anxiety		1614	63.0%
Depression		1486	58.0%
Dyspnea		935	36.5%
Lack of appetite		840	32.8%
Nausea		499	19.5%
General wellbeing		2238	87.3%

* Formally collected as of September 2019. ** ESAS score of 1 or higher.

**Table 5:** Health-related fitness characteristics at baseline (*n* = 2570).

Health-Related Fitness Measures	Total Cohort (*n* = 2570)	
	Mean/No.	SD/%
Body mass index (kg/m^2^)	28.0	6.0
Body mass index category		
Underweight, *n*, %	40	1.5%
Normal, *n*, %	836	32.5%
Overweight, *n*, %	857	33.3%
Obese Class I or II (30–39.9), *n*, %	672	26.1%
Obese Class III or higher (≥40), *n*, %	165	6.4%
Handgrip average score, kg (*n* = 2101)	63.2	18.9
30 s sit-to-stand, no. (*n* = 2570)	14.2	5.3
6 MWT distance, m (*n* = 2101)	539.4	110.2
6 MWT: hallway, m (20–30 m) (*n* = 780)	502.1	113.0
6 MWT: hallway loop, m (*n* = 441)	551.4	99.9
6 MWT: track, m (*n* = 880)	566.5	105.8
Shoulder flexion ROM right, degrees (*n* = 2550)	148.2	13.5
Shoulder flexion ROM left, degrees (*n* = 2552)	146.9	13.7
Single-foot balance: right leg, secs (*n* = 2426)	27.4	17.3
Single-foot balance: left leg, secs (*n* = 2428)	27.0	17.2
2 min step test, no. (*n* = 455)	75.4	22.3

6 MWT—six-minute walk test; m—meters; ROM—range of motion.

## Data Availability

The data presented in this study are available on request from the corresponding author due to ongoing data collection and analyses.

## Supplementary Materials

The following supporting information can be downloaded at: https://www.mdpi.com/article/10.3390/cancers17050772/s1, Table S1: TiDIER Checklist; Table S2: Baseline completion rates for in-person and virtual fitness testing; Table S3: Baseline patient-reported outcomes: Dimensions of Health, Quality of Life, and Function.
