# Supplementary material for: Baseline Characteristics of Participants in the Alberta Cancer Exercise Hybrid Effectiveness–Implementation Study: A Wake-Up Call for Action
Source: Cancers (Basel). 2025 Feb 24;17(5):772. doi: 10.3390/cancers17050772 (PMC11898868; doi:10.3390/cancers17050772)
Supplement: Supplementary file 1 [file cancers-17-00772-s001.zip › cancers-3481855-supplementary.pdf]

Supplementary Table S1: ACE Intervention description using the template for description and replication checklist (TIDier).

Intervention: Alberta Cancer Exercise hybrid effectiveness-implementation study	
Why	ACE aimed to deliver and evaluate high quality, timely, and personalized exercise for the individual after a cancer diagnosis
What: materials	Participants took part in a full body exercise program including aerobic, resistance, balance, and flexibility exercises<br><br>Education included behavior change strategies and motivational tips to increase overall physical activity levels and to support long-term exercise adoption
What: procedures	
Providers	HUB site Clinical Exercise Physiologists (CEPs), with expertise and experience working in the cancer clinical environment, conducted the screening, triage and exercise testing; provided educational support to participants; responsible for oversight of ACE community programming within their hub<br><br>Community-based exercise specialists received cancer-specific training: delivered the 12-week exercise program at the site/virtually<br><br>CEPs/Physical therapists with expertise in cancer supervised/assisted with tailored exercise regimens, as needed, for complex and high-risk participants
How	Supervised classes were delivered in a group circuit-type class in a studio/virtual setting or via group personal training sessions in the site's fitness centre
Where	Community-based cancer exercise facilities: six YMCAs, six municipal fitness centres, three Wellspring Alberta locations (a non-profit cancer support organization), and three academic fitness facilities (University of Calgary, University of Alberta and Lethbridge College)<br><br>Virtual programs were introduced during the COVID-19 pandemic: delivered from the two hub sites at the University of Alberta (Edmonton and Grande Prairie), University of Calgary, and Red Deer Municipal Fitness Facility
Type	Supervised exercise
Intensity	Low-to-moderate intensity exercise: 3 metabolic equivalent units (METS) per session progressed to 5 METS over the 12-week period (progression towards guideline levels of physical activity)
Frequency	Twice weekly
Session time	60-75 minutes per session
Overall duration	12 weeks (follow-ups @ 24 weeks and one year)
Tailoring	Adaptations to address symptoms, cancer-specific impairments, and to reduce risk for adverse effects
Program Fidelity	CEPs provided oversight to ensure fidelity of program: on site day 1 and 24 of program; scheduled monitoring visits and check-ins<br>Community exercise specialists and hub CEPs: Monitoring of symptoms and recording of minor and serious adverse events<br>Outputs: Attendance for number and duration of completed sessions

Supplementary Table S2: Baseline completion rates for in-person and virtual fitness testing.

Baseline Completion Rates	Total Cohort (n = 2570)	ACE North (n = 1299)	ACE South (n = 1271)
In-Person Assessment	(n =2112)	(n =1133)	(n =979)
Questionnaire completion	2107 (99.8%)	1131 (99.8%)	976 (99.7%)
Handgrip strength	2098 (99.3%)	1122 (99.0%)	975 (99.6%)
Shoulder ROM	2095 (99.2%)	1123 (99.1%)	972 (99.3%)
Sit-to-stand	2096 (99.2%)	1125 (99.3%)	971 (99.2%)
Six-Minute Walk Test	2103 (99.6%)	1131 (99.8%)	972 (99.3%)
Single-foot balance	1971 (93.3%)	1051 (92.8%)	945 (96.5%)
Sit-and-reach (optional)	2060 (97.5%)	1114 (98.3%)	946 (96.6%)
Bench Press (optional)	662 (31.3%)	662 (58.4%)	-
Leg Press (optional)	607 (28.7%)	607 (53.6%)	-
Plank endurance (optional)	830 (39.3%)	830 (73.3%)	-
Virtual Assessment	(n = 458)	(n = 166)	(n = 292)
Questionnaire completion	458 (100%)	166 (100%)	292 (100%)
Shoulder ROM	455 (99.3%)	165 (99.4%)	290 (99.3%)
Sit-to-stand	455 (99.3%)	166 (100%)	289 (99.0%)
Two-Minute Step Test	455 (99.3%)	166 (100%)	289 (99.0%)
Single-foot balance	455 (99.3%)	165 (99.4%)	289 (99.0%)
Sit-and-reach (optional)	417(91.0%)	166 (100%)	283 (96.9%)
Plank endurance (optional)	145 (31.7%)	141 (85.0%)	4 (1.3%)

Supplementary Table S3: Baseline patient-reported outcomes: Dimensions of Health, Quality of Life, and Function.

**Cancer and Treatment Characteristics**	**Total Cohort (*n* = 2560)**	
	**Mean/n**	**SD/ %**
EuroQol EQ5D-5L scores		
Mobility (1–5)[a]	1.49	0.75
Self-care (1–5)[a]	1.11	0.39
Usual Activities (1–5) [a]	1.95	0.92
Pain/Discomfort (1–5) [a]	2.04	0.80
Anxiety/Depression (1–5) [a]	1.88	0.83
Self-rated Health Scale (VAS: 0–100) [b]	65.7%	17.8
Functional Assessment of Cancer Therapy (FACT)[b]		
FACT-G [b]	75.8	15.1
Fatigue Subscale [b]	34.8	10.9
RAND 36 Item Health Survey (*n* = 1745) [b]		
Physical Functioning	69.1	21.7
Role Limitations: physical health	39.4	41.3
Role Limitations: emotional problems	60.0	42.2
Energy/Fatigue	49.6	21.3
Emotional Well-being	72.4	17.7
Social Functioning	69.0	23.9
Pain	69.4	23.1
General Health	57.5	20.1
Upper Extremity Functional Scale (*n* = 253) [b] **	69.8	10.4
Lower Extremity Functional Scale (*n* = 272) [b] **	63.5	15.1

[a] lower scores = better functioning; [b] higher scores = better functioning;
*Administered 2017-2020; **Administered during COVID-19 2020-2021
